# Dietary Patterns in Pregnancy and Biomarkers of Oxidative Stress in Mothers and Offspring: The NELA Birth Cohort

**DOI:** 10.3389/fnut.2022.869357

**Published:** 2022-04-12

**Authors:** Eva Morales, Azahara M. García-Serna, Elvira Larqué, María Sánchez-Campillo, Ana Serrano-Munera, Carmen Martinez-Graciá, Marina Santaella-Pascual, Clara Suárez-Martínez, Jesús Vioque, José A. Noguera-Velasco, Francisco V. Avilés-Plaza, Miriam Martínez-Villanueva, Carmen Ballesteros-Meseguer, Lina Galdo-Castiñeira, Luis García-Marcos

**Affiliations:** ^1^Biomedical Research Institute of Murcia (IMIB-Arrixaca), Murcia, Spain; ^2^Department of Public Health Sciences, Faculty of Medicine, University of Murcia, Murcia, Spain; ^3^Department of Biochemistry and Molecular Biology B and Immunology, Faculty of Medicine, University of Murcia, Murcia, Spain; ^4^Department of Physiology, Faculty of Biology, University of Murcia, Murcia, Spain; ^5^Department of Food Science and Technology, Faculty of Veterinary, University of Murcia, Murcia, Spain; ^6^Health and Biomedical Research Institute of Alicante, University Miguel Hernandez (ISABIAL-UMH), Alicante, Spain; ^7^Spanish Consortium for Research on Epidemiology and Public Health (CIBERESP), Madrid, Spain; ^8^Molecular Therapy and Biomarkers Research Group, Clinical Analysis Service, Virgen de la Arrixaca University Clinical Hospital, University of Murcia, Murcia, Spain; ^9^Obstetrics & Gynecology Service, Virgen de la Arrixaca University Clinical Hospital, University of Murcia, Murcia, Spain; ^10^Paediatric Allergy and Pulmonology Units, Virgen de la Arrixaca University Children‘s Hospital, University of Murcia, Murcia, Spain; ^11^ARADyAL Allergy Network, Madrid, Spain

**Keywords:** birth cohort, DASH, dietary indices, oxidative stress, pregnancy, rMED

## Abstract

**Background:**

Although adherence to the Mediterranean and antioxidant-rich diets during pregnancy is suggested to improve maternal-fetal health by reducing oxidative stress, yet there is no study available.

**Objective:**

We examined whether maternal dietary patterns in pregnancy impact the biomarkers of oxidative stress in mothers and their offspring.

**Methods:**

Study population included 642 mothers and 335 newborns of the “Nutrition in Early Life and Asthma” (NELA) birth cohort. Maternal diet during pregnancy was assessed by a validated food frequency questionnaire and *a priori*-defined dietary indices (relative Mediterranean Diet [rMED], alternative Mediterranean Diet [aMED], Dietary Approach to Stop Hypertension [DASH], Alternate Healthy Index [AHEI], and AHEI-2010) were calculated. Biomarkers measured were: hydroperoxides, carbonyl groups, and 8-hydroxydeoxyguanosine (8OHdG) determined in maternal blood and newborn cord blood, and urinary maternal and offspring 15-F2t-isoprostane. Multivariate linear regression models were performed.

**Results:**

Maternal rMED score was inversely associated with the maternal levels of 8OHdG at mid-pregnancy (beta per 1-point increase = −1.61; 95% *CI* −2.82, −0.39) and the newborn levels of hydroperoxides (beta per 1-point increase = −4.54; 95% *CI* −9.32, 0.25). High vs. low maternal rMED score was marginally associated with the decreased levels of 8OHdG in newborns (beta = −9.17; 95% *CI* −19.9, 1.63; *p* for trend 0.079). Maternal DASH score tended to be inversely associated with maternal urinary 15-F2t-isoprostane (beta per 1-point increase = −0.69; 95% *CI*, −1.44, 0.06). High vs. low maternal AHEI score was associated with reduced offspring urinary levels of 15-F2t-isoprostane (beta = −20.2; 95% *CI* −38.0, −2.46; *p* for trend 0.026).

**Conclusion:**

These results suggest that maternal adherence to healthy dietary patterns during pregnancy may reduce DNA damage and lipid oxidation in mothers and offspring.

## Introduction

Diverse exposures or a deficiency of antioxidant systems lead to an unregulated production and accumulation of reactive oxygen species (ROS), which results in the increase oxidative stress (1). The interaction of ROS with proteins, lipids, carbohydrates, and nucleic bases leads to the formation of diverse biomarkers of oxidative stress which include hydroperoxides and isoprostanes (lipid oxidation), carbonyl groups (protein oxidation), and 8-hydroxy-deoxyguanosine (8OHdG) (DNA damage), that impair normal cellular and tissue functions.

Oxidative stress balance in fetal life can be considered one facet of the Developmental Origins of Health and Disease hypothesis. Embryo development occurs in a relatively low-oxygen environment and is highly sensitive to the injury to oxidant molecules because of its low antioxidant capacity (2). Oxidative stress is associated with the generation of ROS, which have both physiologic and pathologic roles in the placenta (3), embryo, and fetus. During pregnancy, oxidative stress may impose adverse effects on mother and child health; it has been involved in the hypoxia of complicated pregnancies, such as pregnancy-induced hypertension and preeclampsia (4), gestational diabetes mellitus (GDM) (5), impaired fetal growth (6), preterm birth (7), small for gestational age (8), and low birth weight (9). Moreover, oxidative stress may be a connecting link between intrauterine environment and programmed health consequences after birth. To this regard, some studies have also revealed the long-term impacts of prenatal oxidative stress on the offspring, such as infant adiposity (10) and the risk of asthma and allergic disease (11, 12).

A well-balanced diet seems to be the main source of non-enzymatic antioxidants. Healthy diets characterized by a high intake of fruit, vegetables, and whole grains as well as healthy fats, such as mono and polyunsaturated fats and a low intake of saturated fat are aggregate sources of bioactive compounds that act as components in antioxidant systems. Evidence from observational and intervention studies has shown that the Mediterranean diet (MD) and the Dietary Approach to Stop Hypertension (DASH) may reduce the concentrations of various biomarkers of oxidative stress and inflammation (13). In observational studies, adherence to the MD pattern was associated with the lower levels of F2-Isoprostane (14) and the higher levels of total antioxidant capacity (TAC) (15) in adult populations. In addition, following an intervention based on MD led to significantly reduced levels of biomarkers reflecting different aspects of oxidative stress, such as biomarkers of lipid peroxidation (F2-isoprostanes) and oxidative DNA damage (8OHdG) in adult subjects (16, 17).

Adherence to healthy dietary patterns (i.e., Mediterranean and antioxidant-rich diets) during pregnancy is suggested to improve maternal health and to facilitate the correct fetal development by reducing oxidative stress levels during the critical windows of susceptibility; however, the relationship between maternal adherence to the healthy dietary patterns during pregnancy and the biomarkers of oxidative stress in pregnant women and their offspring has not been evaluated yet. Therefore, we sought to address whether maternal adherence to healthy dietary patterns during pregnancy is associated with the concentrations of biomarkers of oxidative stress measured in mothers and their offspring.

## Materials and Methods

### Study Design and Participants

The study population were the mother-child pairs of the Nutrition in Early Life and Asthma (NELA) study, a prospective population-based birth cohort set up between 2015 and 2018 in Murcia, a south-eastern Mediterranean region of Spain. The main objective of NELA is to unravel the developmental origins and mechanisms of asthma and allergy. The study protocol, recruiting methods, and data collection processes have been described elsewhere (18). Among the 1,350 women invited to participate, 738 (54%) were finally enrolled in the study. The selection of participants of the present study is presented in Supplementary Figure 1.

The study protocol was reviewed and approved by the Ethics Committee of the Virgen de la Arrixaca University Clinical Hospital in accordance with the guidelines of the Declaration of Helsinki (report 9/14; 29/09/2014). Written informed consents were obtained from parents at recruitment.

### Maternal Dietary Intake Assessment and Dietary Patterns During Pregnancy

Information on the eating habits of pregnant women was collected at 20 weeks of gestation to estimate dietary intakes during that period using a semi-quantitative food frequency questionnaire (FFQ) previously developed and validated for use among pregnant women living in Spain (19). The FFQ included 123 items, of which 112 were semi-quantitative and 11 qualitative to collect information about the use of supplements and organic food consumption. The intakes of nutrients and energy were estimated using the U.S. Department of Agriculture Food Composition Tables (20), and as well as other published sources for Spanish foods, portion sizes, and their content for some specific nutrients, such as folic acid (21, 22). The intake frequency for each food item was converted to the average daily intake for each participant. For the calculation of the different scores in each of the dietary patterns described below, the consumption of vitamin or mineral supplements by the mother during pregnancy was not considered.

Based on the FFQ data, we calculated five of the most widely used *a priori*-defined dietary indexes that we have used previously (23): the relative Mediterranean Diet (rMED) and the alternative Mediterranean Diet (aMED) indices that are based on the Mediterranean dietary pattern; the Dietary Approaches to Stop Hypertension (DASH) index; and the Alternate Healthy Index (AHEI) and its version for evaluating chronic diseases (AHEI-2010), which are based on American dietary guides.

The rMED and aMED indices are the versions of the original MD score developed by Trichopulou et al. (24). The rMED was constructed considering the consumption of eight components: vegetables, fruits and nuts, cereals, legumes, fish, olive oil, meat, and dairy products (25). To adapt to the score of pregnant women, the component on alcohol consumption was removed because the recommendation is to avoid alcohol consumption during pregnancy and most of the women in our cohort did not consume it. All the food groups were measured as grams per 1,000 kcal/day, and values were divided into tertiles. We assigned values of 0, 1, and 2 to the intake tertiles, positively scoring higher intakes for the 6 components that fit into the MD. The scoring was reversed for meat and dairy components presumed to not fit into the MD, thus positively scoring lower intakes. Scores were added for each component, up to a total score ranging from 0 to 16. Differently from rMED, aMED considers red and processed meat and establishes the ratio of mono/polyunsaturated fats (26) and determines a score ranging from 0 to 8.

The DASH index was constructed based on the DASH clinical trial in which dietary pattern rich in fruit, vegetables, and low-fat dairy products can substantially lower blood pressure (27). This *a priori*-defined index was described by Fung et al. in 2008 (28). The DASH index was constructed considering the consumption of eight components, such as fruits, vegetables, nuts and legumes, whole grains, low-fat dairy, sodium, red and processed meats, and sweetened beverages. We assigned values of 1, 2, 3, 4, or 5 to the intake quintiles, positively scoring higher intakes for all the components except for sodium, red and processed meats, and sweetened beverages which had a reverse scoring (positively scoring lower intakes). Scores were summed for each component, for a total score ranging 0–40.

The AHEI is an *a priori* dietary index that is based on the Healthy Eating Index, which was developed by the U.S. Department of Agriculture and considers the adherence to the food guide pyramid. The AHEI is a measure of diet quality that focuses on foods and macronutrients, such as assessment of unsaturated fats, associated with decreased chronic disease risk (29). Briefly, the AHEI consists of 9 items with a maximum possible score of 87.5. Such items and their corresponding ideal intakes are as follows: vegetables (5 servings/day), fruit (4 servings/day), nuts and soy (1 serving/day), ratio of white meat (fish and poultry) to red meat (≥4:1), cereal fiber (15 g/day), trans fat ( ≤ 0.5% of energy), ratio of polyunsaturated to saturated fat (≥1), moderate alcohol intake (0.5–1.5 servings/day), and long-term multivitamin use (≥5 years of continuous use). To make the index more appropriate for our pregnant population, we excluded the “long-term multivitamin use” item because it is not a common practice in the Spanish population. Scores were summed for each component, for a total score ranging 0–80.

The AHEI-2010 index was designed in 2012 based on updated literature to study the relation between food intake and chronic diseases (30). The AHEI-2010 scores 11 components for a total of 110 points, such as whole grains intake (specific for women), legumes and nuts, red/processed meat ratio, sugar-sweetened beverages and fruit juices, sodium, and polyunsaturated fats.

Definition of the scoring system, number of components, total score, and components of dietary scores are presented in Supplementary Table 1.

### Biological Samples and Measurement of Biomarkers of Oxidative Stress

Mother fasting blood and urine samples were collected once after 12 h overnight fast at mid-pregnancy (20–24 weeks of gestation). Offspring venous cord blood samples were collected at birth and spot urine samples were collected at 3 months of age. All samples were processed within 48 h and stored at −80°C until analysis. Hemolyzed samples were excluded.

#### Determination of Plasma Carbonyl Groups

The plasma levels of carbonyl groups as a measurement of protein oxidation was determined by 2,4-dinitrophenylhydrazine (DNPH) reagent according to the method of Reznick and Packer (31).

#### Determination of Plasma Hydroperoxides

Serum hydroperoxide levels were analyzed by the method described by Jiang et al. (32) using a ferrous oxidation/xylenol orange (FOX) method.

#### Urinary Levels of Isoprostanes

The concentrations of 15-Isoprostanes F2 in urine were quantified using a urinary 8-isoprostane competitive enzyme-linked immunosorbent assay (ELISA) kit (Oxford Biomedical Research Inc., Oxford, MI). Intra- and inter-assay CV was ≤ 10%. The values were normalized per milligram of creatinine. Creatinine was assayed using automated system (Cobas c 702, Roche Diagnostics, Mannhein, Germany). Inter-assay CV was ≤ 2.5%. All isoprostane concentrations were adjusted to account for urinary dilution by dividing isoprostane concentrations (ng/ml) by creatinine levels (mg/dl) with results reported in ng/mg creatinine.

#### 8OHdG

DNA oxidation in the serum of mothers and their offspring was estimated by the measurement of 8-Hydroxy-2'-deoxyguanosine (8-OHdG) with an ELISA kit (Japan Institute for the Control of Aging, Fukuroi, Shizuoka, Japan). Intra- and inter-assay CV were ≤ 8%.

### Covariates

The following variables were considered as potential confounder factors because of their possible associations with the levels of biomarkers of oxidative stress and dietary patterns in pregnancy. We obtained information through questionnaires administered in person during pregnancy, such as maternal age, social class (using a widely used Spanish adaptation of the international ISCO88 coding system: I–II, managers/technicians; III, skilled; IV–V, semiskilled/unskilled; and unemployed) (33); educational level (incomplete secondary or less, complete secondary, and university); parity (0, nulliparous; *vs*. 1 or more, no nulliparous); maternal pre-pregnancy body mass index (BMI) based on height and pre-pregnancy self-reported weight (kg/m^2^) (categorized as normal BMI <25, overweight 25 < BMI <30, and obesity BMI ≥ 30); maternal smoking during pregnancy (yes/no); self-reported physical activity during pregnancy (sedentary, poorly active, moderately active and strongly active); paracetamol use during pregnancy (yes/no); gestational diabetes mellitus (yes/no); hypertension during pregnancy (yes/no); total intake of calories (kcal/day); and alcohol intake (g/day). Information related to child's sex, birthweight (g); gestational age (weeks); mode of delivery (vaginal non-instrumental, vaginal instrumental, and cesarean section); oxytocin use (yes/no); and Apgar score at 5th min was obtained from clinical records. The season of birth (spring, March–May; summer, June–August; fall, September–November; and winter, December–February) was also considered.

### Statistical Analysis

We described the baseline characteristics of participants using means and standard deviation (SD) (normally distributed continuous variables), medians and interquartile range (IQR) (non-normally distributed continuous variables), and numbers and proportions (categorical variables). Comparisons between groups were tested by Student's *t*-tests or Kruskal–Wallis tests, and Pearson's chi-squared tests.

Multiple linear regression models were used to estimate the beta coefficients for the association between maternal dietary indices in pregnancy (expressed both in tertiles and as continuous variables per 1-point increment) and the concentrations of biomarkers of oxidative stress measured in mothers and their offspring. The concentrations of biomarkers of oxidative stress showed non-normal distributions and were transformed using the natural log (ln) before analysis. The measures of associations are presented as log-transformed mean differences and regression coefficients, which can be interpreted as percentages after multiplying by 100 (34), or as geometric mean differences with their 95% confidence interval (*CI*). Only covariates that were associated both with the exposure and the outcome of interest were used for adjustment, following the backward stepwise method (*p* < 0.1). The final covariates included in the models were maternal social class, maternal smoking during pregnancy, and the total intake of calories. For all the models, we calculated the value of *p* for trend using the exposure variable in tertiles as continuous.

Linear dose–response relation between continuous maternal dietary scores and the concentrations of biomarkers of oxidative stress was assessed using adjusted generalized additive models (GAMs) by the graphical examination and likelihood ratio test (35). GAMs indicated that the associations between dietary indexes and biomarkers did not deviate from linearity (*p*-gain > 0.10).

Data were analyzed in Stata Software (version 15.1, StataCorp, College Station, Texas, USA).

## Results

The main characteristics of study participants are summarized in Table 1. Mother sampled in mid-pregnancy and their offspring showed similar characteristics to the full cohort. The mean (± SD) age of included women was 32.7 (±4.5) years, 49% were primiparous and 55% had high educational level. Overall, 21% of women were overweight and 9% obese before pregnancy, median gestational weight gain was 12 kg, and 40% of women reported to be moderately or strongly active during pregnancy. Overall, 15.7% of women reported to smoke and 64% to use paracetamol during pregnancy. The prevalence of gestational diabetes and hypertension in pregnancy was 7.8 and 2.6%, respectively. The rate of cesarean section was 21%. The study population included 52% male newborns. Median gestational age was 39.8 weeks, 2.6% of newborns were premature and 4.3% had low birthweight.

**Table 1:** Baseline characteristics of the subjects available for maternal dietary patterns in pregnancy and oxidative stress biomarkers measurement at mid-pregnancy (*n* = 642) and at birth (*n* = 335). The Nutrition in Early Life and Asthma (NELA) study.

	**N**	**Full cohort (*n* = 738)**	**N**	**Mid-pregnancy sampled participants (*n* = 642)**	**N**	**Subjects with cord blood measurements (*n* = 335)**
**Maternal age, years, mean** **±SD**	738	32.6 ± 4.6	642	32.7 ± 4.5	335	32.9 ± 4.3
**Maternal education level, n (%)**	738		642		335	
Incomplete secondary or less		146 (19.8)		120 (18.7)		61 (18.2)
Complete secondary		191 (25.9)		168 (26.2)		79 (23.6)
University		401 (54.3)		354 (55.1)		195 (58.2)
**Maternal social class**, ***n*** **(%)**	738		642		335	
I-II (managers/technicians)		264 (35.8)		234 (36.5)		129 (38.5)
III (skilled)		167 (22.6)		143 (22.3)		85 (25.4)
IV-V (semiskilled/unskilled)		144 (19.5)		131 (20.4)		55 (16.4)
Unemployed		163 (22.1)		134 (20.9)		66 (19.7)
**BMI before pregnancy (kg/m** ^**2**^ **), median (IQR)**	731	23.0 (20.9, 25.7)	638	23.0 (20.8, 25.9)	333	23.2 (21.1, 26.4)
Normal (<25)	509	509 (69.9)	442	442 (69.3)	216	216 (64.9)
Overweight (25–29.99)	160	160 (21.9)	137	137 (21.5)	86	86 (25.8)
Obese (≥30)	62	62 (8.5)	59	59 (9.2)	31	31 (9.3)
**Parity, nulliparous**, ***n*** **(%)**	738	374 (50.7)	642	316 (49.2)	335	163 (48.7)
**Maternal smoking in pregnancy, yes, (** ***n*** **) %**	738	128 (17.3)	642	101 (15.7)	335	54 (16.1)
**Physical activity in pregnancy**, ***n*** **(%)**	738		642		335	
Sedentary		119 (16.1)		103 (16.0)		64 (19.1)
Poorly active		324 (43.9)		285 (44.4)		144 (43.0)
Moderately active		264 (35.8)		226 (35.2)		117 (34.9)
Strongly active		31 (4.2)		28 (4.4)		10 (3.0)
**Paracetamol use in pregnancy, ever**, ***n*** **(%)**	673	429 (63.7)	602	387 (64.3)	322	215 (66.8)
**Gestational DM, yes**, ***n*** **(%)**	710	58 (8.2)	629	49 (7.8)	335	29 (8.7)
**Hypertension in pregnancy, yes**, ***n*** **(%)**	706	18 (2.6)	624	16 (2.6)	335	9 (2.7)
**Gestational weight gain, median (IQR)**	678	12 (9.2, 15.0)	599	12 (9.0, 15.0)	327	12 (9, 15.0)
**Type of delivery**, ***n*** **(%)**	712		629		335	
Vaginal non-instrumental		409 (57.4)		365 (58.0)		192 (57.3)
Vaginal Instrumental		146 (20.5)		134 (21.3)		71 (21.2)
Cesarean section		157 (22.1)		130 (20.7)		72 (21.5)
**Oxytocin use, yes**, ***n*** **(%)**	619	480 (77.5)	545	420 (77.1)	319	238 (74.6)
**Newborn sex, male**, ***n*** **(%)**	720	357 (49.6)	633	314 (49.6)	335	176 (52.5)
**Gestational age, median (IQR)**	720	39.8 (38.8, 40.6)	633	39.8 (38.8, 40.7)	335	39.8 (39.1, 40.7)
**Preterm (<37 weeks), yes**, ***n*** **(%)**	720	36 (5.0)	633	31 (4.9)	335	12 (3.6)
**Season of birth**, ***n*** **(%)**	720		633		335	
Autumn (September-November)		202 (28.1)		184 (29.1)		112 (33.4)
Spring (March-May)		186 (25.8)		158 (24.9)		72 (21.5)
Summer (June-August)		208 (28.9)		186 (29.4)		98 (29.3)
Winter (December-February)		124 (17.2)		104 (16.6)		53 (15.8)
**Birthweight (g), mean** **±SD**	712	3,242.2 ± 474.8	629	3,252.8 ± 463.8	335	3,287.0 ± 435.1
**Low birthweight (<2500 g), yes, n (%)**	712	42 (5.9)	629	35 (5.6)	335	14 (4.2)
**Apgar score (5th min), mean** **±SD**	710	9.9 ± 0.3	628	9.9 ± 0.4	335	9.9 ± 0.4
**Maternal calories intake (kcal/day)**	665	2,139.2 ± 642.7	642	2,142.3 ± 645.4	335	2,097.8 ± 604.5
**Maternal alcohol intake (g/day)**	665	0.17 ± 0.52	642	0.17 ± 0.52		0.18 ± 0.56
**Dietary patterns in pregnancy, median (IQR)**						
Relative Mediterranean Diet Score (rMED)	665	8 (6,10)	642	8 (6,10)	335	8 (6,10)
Alternate Mediterranean Diet (aMED)	665	4 (3,5)	642	4 (3,5)	335	4 (3,5)
Dietary Approach to Stop Hypertension (DASH)	665	24 (20,27)	642	24 (20,27)	335	24 (20,27)
Alternate Healthy Eating Index (AHEI)	665	43 (38,49)	642	43 (38,49)	335	43 (38,48)
Alternate Healthy Eating Index 2010 (AHEI-2010)	665	61 (55,65)	642	61 (55,65)	335	60 (56,65)

Median and IQR values for maternal rMED, aMED, DASH, AHEI, and AHEI-2010 indices during pregnancy were 8 (IQR: 6–10.0), 4 (IQR: 3–5), 24 (IQR: 20–27), 43 (IQR: 38–49), and 61 (IQR: 55–65), respectively (Table 1). Maternal dietary indices in pregnancy were modestly to highly correlated with each other, having a Spearman's rho correlation coefficients between 0.23 and 0.76 (Supplementary Table 2).

Mothers with higher adherence to rMED, DASH, AHEI, and AHEI-2010 were older, had higher educational level and social class, and tended to be non-smokers during pregnancy as compared with mothers with low adherence to these dietary patterns (Supplementary Tables 3–7). Mothers with higher adherence to rMED and DASH had a lower use of paracetamol during pregnancy than those with low adherence to these dietary patterns (Supplementary Tables 3, 5). Nulliparous mothers had higher adherence to DASH but lower adherence to AHEI-2010 than those non nulliparous (Supplementary Tables 5, 7). Mothers with higher adherence to DASH and AHEI tended to be more physically active. Mother with gestational diabetes mellitus had higher adherence to rMED. Lower gestational weight gain was associated with the higher adherence to AHEI-2010 (Supplementary Table 7).

The mothers and offspring levels of biomarkers of oxidative stress are presented in Table 2. The mean levels of carbonyl groups were lower in mothers at mid-pregnancy than in their neonates. On the contrary, the mean circulating levels of hydroperoxides and 8OHdG and as well as the mean levels of urinary isoprostanes were higher in mothers compared with their offspring.

**Table 2:** Distributions of the biomarkers of oxidative stress in mothers and their offspring, the NELA study.

**Biomarker**	**N**	**Min**	**P25**	**Mean ±SD**	**P50**	**P75**	**Max**
Mothers^*^							
Carbonyl groups (nmol/mg)	642	0.024	0.032	0.036 ± 0.006	0.036	0.040	0.079
Hydroperoxides (nmol/mL)	642	0.0	0.14	0.24 ± 0.14	0.23	0.30	1.11
Urine 15-F2t-isoprostane (ng/mg creatinine)	615	0.11	2.76	4.23 ± 2.20	3.79	5.17	17.9
8OHdG (ng/ml)	543	5.32	8.93	11.9 ± 7.3	10.4	12.3	103.3
Offspring^**^							
Carbonyl groups (nmol/mg)	335	0.026	0.038	0.043 ± 0.008	0.042	0.048	0.084
Hydroperoxides (nmol/mL)	333	0.0	0.024	0.093 ± 0.093	0.071	0.126	0.719
Urine 15-F2t-isoprostane (ng/mg creatinine)	462	0.53	1.29	3.28 ± 2.83	1.81	5.04	14.48
8OHdG (ng/ml)	243	3.36	7.23	9.64 ± 5.24	8.64	10.26	55.71

* *All biomarkers were measured in maternal blood or urine collected mid-pregnancy*.

** *All biomarkers were measured in cord blood of newborns at birth, except 15-F2t-isoprostane that was measured in offspring's urine at 3 months of age*.

**Figure 1:**
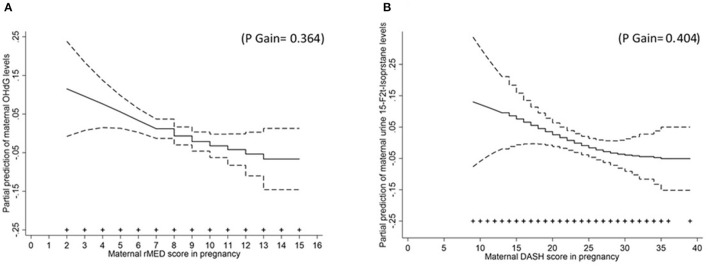
The relation (and 95% confidence levels [*CI*s]) of maternal dietary scores in pregnancy with the concentrations of selected maternal biomarkers of oxidative stress. General additive models adjusted for maternal social class, maternal smoking during pregnancy, and total intake of calories for **(A)** the maternal relative Mediterranean Diet Score (rMED) score and circulating levels of 8-hydroxydeoxyguanosine (8OHdG); and **(B)** maternal Dietary Approach to Stop Hypertension (DASH) score and urinary isoprostanes levels. The symbols (+) on the *X*-axis indicate maternal dietary score observations.

**Figure 2:**
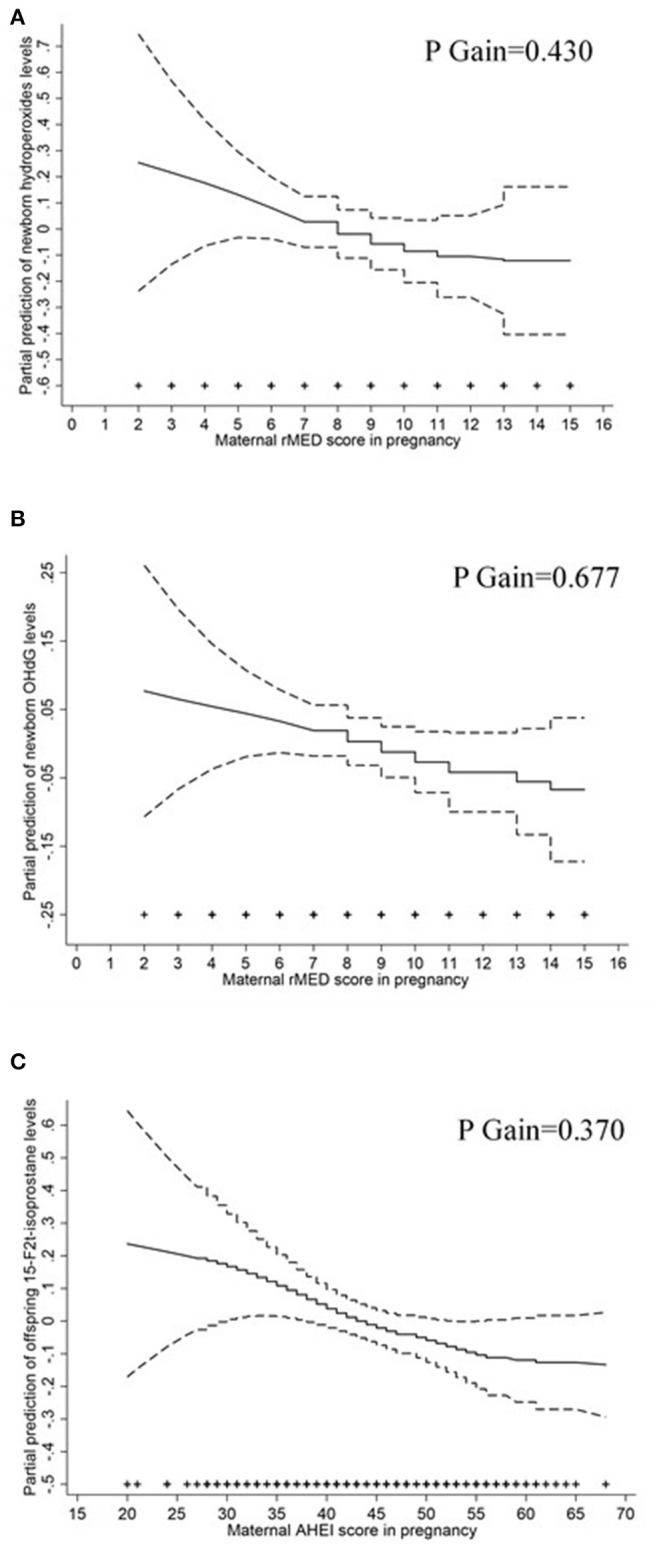
The relation (and 95% *CI*s) of maternal dietary scores in pregnancy with the concentrations of selected offspring biomarkers of oxidative stress. General additive models adjusted for maternal social class, maternal smoking during pregnancy, and total intake of calories for **(A)** maternal rMED score and circulating levels of hydroperoxides in newborns; and **(B)** maternal rMED score and circulating levels of 8OHdG in newborns; and **(C)** maternal Alternate Healthy Index (AHEI) score and offspring urinary 15F-2t-isoprostane levels. The symbols (+) on the *X*-axis indicate maternal dietary score observations.

After adjustment for potential cofounders, we found that high vs. low maternal adherence to rMED score during pregnancy was associated with the reduced circulating maternal levels of 8OHdG in mid-pregnancy (beta = −8.02; 95% *CI* −15.4, −0.64; *p* for trend = 0.026) showing a linear relationship (*p* gain = 0.364) (Figure 1A) and a reduction per 1-point increase of rMED score of −1.61 (95% *CI* −2.82, −0.39) (Table 3). Moreover, maternal rMED score during pregnancy tended to be inversely related to the offspring levels of circulating hydroperoxides at birth (beta per 1-point increase = −4.54; 95% *CI* −9.32, 0.25) showing a linear relationship (*p* gain = 0.430) (Figure 2A). High vs. low maternal adherence to rMED score during pregnancy was marginally associated with the reduced offspring levels of 8OHdG at birth (beta = −9.17; 95% *CI* −19.9, 1.63; *p* for trend 0.079) (Table 3), showing a linear relationship (*p* gain = 0.677) (Figure 2B). There were no associations between maternal aMED score during pregnancy and the levels of biomarkers of oxidative stress either in mothers or their offspring (Table 3).

**Table 3:** Associations between maternal rMED and aMED indices in pregnancy and the biomarkers of oxidative stress in mothers and their offspring. The NELA study.

	**rMED**				
	**T1 (2–7)**	**T2 (8–9)**	**T3 (10–15)**		**Per 1–point increase**
		**β (95% CI)**	**β (95% CI)**	**P for trend**	**β (95% CI)**	
**Mothers**						
Carbonyl groups	Ref.	3.25 (0.25, 6.24)	2.27 (−0.74, 5.27)	0.101	0.48 (−0.01, 0.97)	
Hydroperoxides	Ref.	−10.9 (−23.6, 1.89)	−7.65 (−20.4, 5.13)	0.194	−1.14 (−3.25, 0.98)	
Urine 15–F2t–isoprostane	Ref.	−4.72 (−13.9, 4.50)	−2.03 (−11.2, 7.15)	0.606	−0.60 (−0.21, 0.92)	
8OHdG	Ref.	−7.62 (−14.9, −0.28)	−8.02 (−15.4, −0.64)	0.026	−1.61 (−2.82, −0.39)	
**Offspring**						
Carbonyl groups	Ref.	−0.20 (−5.01, 4.61)	1.09 (−5.01, 4.61)	0.670	0.02 (−0.62, 0.90)	
Hydroperoxides	Ref.	−11.8 (−41.4, 17.8)	−22.1 (−51.6, 7.43)	0.138	−4.54 (−9.32, 0.25)	
Urine 15–F2t–isoprostane	Ref.	−3.97 (−22.0, 14.1)	−10.5 (−27.8, 6.80)	0.236	−2.35 (−5.19, 0.49)	
8OHdG	Ref.	−9.88 (−21.1, 1.36)	−9.17 (−19.9, 1.63)	0.079	−1.99 (−3.14, 0.37)	
	**aMED**				
	**T1 (0–3)**	**T2 (4,5)**	**T3 (6-8)**		**Per 1–point increase**
		**β** **(95% CI)**	**β** **(95% CI)**	**P for trend**	**β** **(95% CI)**	
**Mothers**						
Carbonyl groups	Ref.	1.02 (−1.73, 3.77)	−0.22 (−3.64, 3.19)	0.935	0.16 (−0.54, 0.86)	
Hydroperoxides	Ref.	2.51 (−9.23, 14.2)	0.10 (−14.5, 14.7)	0.901	−0.30 (−3.27, 2.66)	
Urine 15–F2t–isoprostane	Ref.	−0.59 (−9.08, 7.90)	−0.25 (−10.8, 10.2)	0.939	−1.48 (−3.63, 0.65)	
8OHdG	Ref.	0.95 (−5.84, 7.75)	−1.77 (−10.1, 6.53)	0.765	0.07 (−1.67, 1.80)	
**Offspring**						
Carbonyl groups	Ref.	1.66 (−2.75, 6.06)	2.20 (−3.28, 7.67)	0.380	0.09 (−1.07, 1.25)	
Hydroperoxides	Ref.	−14.5 (−41.6, 12.7)	−17.2 (−51.8, 17.4)	0.258	−1.50 (−8.86, 5.86)	
Urine 15-F2t-isoprostane	Ref.	−3.21 (−19.5, 13.1)	−15.7 (−35.2, 3.75)	0.135	−2.96 (−6.99, 1.07)	
8OHdG	Ref.	1.69 (−8.69, 12.1)	−2.41 (−14.8, 10.0)	0.773	−0.31 (−2.98, 2.35)	

*rMED score: range 1–15, in 3 tertiles, to define: low (T1), medium (T2), and high (T3) adherence to the Mediterranean diet (MD). aMED score: range 0–8, in 3 tertiles, to define: low (T1), medium (T2), and high (T3) adherence to the MD. All models are adjusted for maternal social class, maternal smoking during pregnancy, and total intake of calories*.

High vs. low maternal adherence to the categorical DASH score during pregnancy was marginally associated with the reduced maternal urinary levels of F2-isoprostanes in mid-pregnancy (beta = −8.31; 95% *CI* −17.9, 1.33; *p* for trend = 0.090) (Table 4) with a liner relationship (*p* gain = 0.404) (Figure 1B) and a reduction per 1-point increase of −0.69 (95% *CI*, −1.44, 0.06). There were no associations between maternal adherence to DASH dietary pattern during pregnancy and the levels of biomarkers in the offspring (Table 4).

**Table 4:** Associations between maternal Dietary Approach to Stop Hypertension (DASH) index in the pregnancy and biomarkers of oxidative stress in mothers and their offspring. The NELA study.

	**DASH**				
	**T1 (9–21)**	**T2 (22–26)**	**T3 (27–39)**		**Per 1–point increase**
		**β (95% CI)**	**β (95% CI)**	**P for trend**	**β (95% CI)**	
**Mothers**						
Carbonyl groups	Ref.	0.82 (−2.21, 3.85)	−0.06 (−3.22, 3.09)	0.979	0.02 (−0.22, 0.27)	
Hydroperoxides	Ref.	5.51 (−7.37, 18.4)	1.55 (−11.9, 14.9)	0.809	0.44 (−0.61, 1.49)	
Urine 15–F2t–isoprostane	Ref.	−4.90 (−14.2, 4.36)	−8.31 (−17.9, 1.33)	0.090	−0.69 (−1.44, 0.06)	
8OHdG	Ref.	1.74 (−5.81, 9.29)	2.95 (−4.82, 10.7)	0.456	0.27 (−0.34, 0.87)	
**Offspring**						
Carbonyl groups	Ref.	−0.69 (−5.59, 4.21)	−2.71 (−7.67, 2.25)	0.280	−0.24 (−0.65, 0.16)	
Hydroperoxides	Ref.	2.82 (−27.9, 33.6)	−3.22 (−34.4, 27.9)	0.831	−0.67 (−0.32, 1.90)	
Urine 15–F2t–isoprostane	Ref.	5.05 (−13.0, 23.1)	−4.05 (−22.4, 14.3)	0.659	−0.59 (−2.02, 0.84)	
8OHdG	Ref.	−0.88 (−12.6, 10.8)	2.20 (−9.23, 13.6)	0.693	0.39 (−0.49, 1.29)	

*DASH score: range 9–39, in 3 tertiles, to define: low (T1), medium (T2), and high (T3) adherence to DASH. All models adjusted for maternal social class, maternal smoking during pregnancy, and total intake of calories*.

We found high vs. low maternal adherence to AHEI score to be associated with the reduced offspring urinary levels of 15-F2t-isoprostane (beta = −20.2; 95% *CI* −38.0, −2.46; *p* for trend = 0.026), with a linear relationship (*p* gain = 0.370) (Figure 2C) and a reduction per 1-point increase of −1.07 (95% *CI* −1.99, −0.14) (Table 5). There were no associations between maternal adherence to AHEI-2010 score during pregnancy and the levels of biomarkers of oxidative stress either in mothers or their offspring (Table 5).

**Table 5:** Associations between maternal Alternate Healthy Index (AHEI) and AHEI-2010 indices in the pregnancy and biomarkers of oxidative stress in mothers and their offspring. The NELA study.

	**AHEI**			
	**T1 (20–40)**	**T2 (41–46)**	**T3 (47–68)**		**Per 1–point increase**
		**β (95% CI)**	**β (95% CI)**	**P for trend**	**β (95% CI)**	
**Mothers**						
Carbonyl groups	Ref.	−1.36 (−4.35, 1.64)	−0.61 (−3.67, 2.44)	0.674	−0.13 (−0.29, 0.03)	
Hydroperoxides	Ref.	−11.1 (−23.8, 1.60)	1.20 (−11.8, 14.1)	0.917	0.09 (−0.60, 0.76)	
Urine 15–F2t–isoprostane	Ref.	−1.59 (−10.8, 7.63)	−6.63 (−16.0, 2.72)	0.167	−0.33 (−0.82, 0.15)	
8OHdG	Ref.	1.38 (−6.11, 8.87)	0.48 (−7.07, 8.03)	0.898	−0.04 (−0.43, 0.36)	
**Offspring**						
Carbonyl groups	Ref.	0.21 (−4.57, 5.00)	−0.56 (−5.48, 4.36)	0.831	−0.11 (−0.37, 0.16)	
Hydroperoxides	Ref.	4.11 (−25.5, 33.7)	−5.07 (−36.6, 26.4)	0.774	−0.78 (−2.53, 0.97)	
Urine 15–F2t–isoprostane	Ref.	−23.0 (−40.9, −5.14)	−20.2 (−38.0, −2.46)	0.026	−1.07 (−1.99, −0.14)	
8OHdG	Ref.	−6.79 (−17.9, 4.33)	−3.63 (−15.1, 7.84)	0.527	−0.30 (−0.90, 0.30)	
	**AHEI−2010**			
	**T1 (42–58)**	**T2 (59–63)**	**T3 (64–83)**		**Per 1–point increase**
		**β** **(95% CI)**	**β** **(95% CI)**	**P for trend**	**β** **(95% CI)**	
**Mothers**						
Carbonyl groups	Ref.	−0.42 (−3.54, 2.70)	0.42 (−2.60, 3.44)	0.790	0.02 (−0.16, 0.21)	
Hydroperoxides	Ref.	6.94 (−6.31, 20.1)	−6.85 (−19.6, 5.93)	0.308	−0.33 (−1.11, 0.44)	
Urine 15–F2t–isoprostane	Ref.	−4.36 (−13.8, 5.14)	−2.24 (−11.5, 7.01)	0.623	−0.30 (−0.87, 0.27)	
8OHdG	Ref.	2.53 (−5.25, 10.3)	−2.44 (−9.83, 4.96)	0.514	−0.20 (−0.65, 0.25)	
**Offspring**						
Carbonyl groups	Ref.	−2.56 (−7.67, 2.55)	0.60 (−4.05, 5.26)	0.794	−0.07 (−0.36, 0.21)	
Hydroperoxides	Ref.	13.6 (−18.2, 45.3)	−17.9 (−47.4, 11.7)	0.235	−1.52 (−3.28, 0.24)	
Urine 15–F2t–isoprostane	Ref.	−6.69 (−25.3, 11.9)	−6.65 (−24.4, 11.1)	0.465	−0.03 (−1.11, 1.06)	
8OHdG	Ref.	5.18 (−6.76, 17.1)	8.57 (−2.42, 19.6)	0.126	0.51 (−0.16, 1.18)	

*AHEI score: range 20–68, in 3 tertiles, to define: low (T1), medium (T2), and high (T3) adherence to the AHEI. AHEI-2010 score: range 42–83, in 3 tertiles, to define: low (T1), medium (T2), and high (T3) adherence to the updated version of the AHEI. All models adjusted for maternal social class, maternal smoking during pregnancy, and total intake of calories*.

## Discussion

To the best of our knowledge, this is the first study that investigates the associations between maternal adherence to different food-based diet quality scores during pregnancy and the biomarkers of oxidative stress in mothers and their offspring. We found that higher maternal adherence to MD pattern, evaluated by the rMED index, decreased maternal oxidative DNA damage measured by 8OHdG levels and offspring lipid peroxidation measured by hydroperoxides levels. Moreover, greater maternal adherence to the DASH pattern was associated with reduced maternal lipid oxidation during pregnancy measured by the urinary levels of 15-F2t-isoprostane. In addition, a higher maternal AHEI score was associated with lower lipid oxidation in the offspring measured by the urinary levels of 15-F2t-isoprostane.

Maternal rMED score, but not aMED score, was related to the reduced levels of maternal 8OHdG at mid-pregnancy, one of the most used markers for assessing DNA damage. Accordingly, other studies conducted in adults did not find an association between aMED index and urinary 8OHdG concentrations (36). Although both indices are the versions of original MD score, rMED index but not aMED considers the consumption of olive oil. To this regard, Luisi et al. (17) have reported the decreased levels of 8OHdG after MD rich in extra virgin olive oil, an essential component of this diet pattern that has a major beneficial role attributed to oleic acid and polyphenols. Both compounds exert antioxidant activity positively influencing several biomarkers of oxidative damage (37). Increased maternal levels of 8OHdG (DNA damage) have been related to the risk of GDM, reduced birthweight, and preterm birth (38, 39). Moreover, the newborns of mothers with higher rMED scores showed the lower levels of hydroperoxides, a marker of lipid oxidation. In this regard, MD characterized by a high intake of vegetables and olive oil provide phenolic and polyphenols that have been found to reduce the generation of lipid hydroperoxides (40). Our results suggest that the reported beneficial effects of adherence to MD during gestation and early postnatal life on pregnancy and offspring growth outcomes (41–43) may be mediated through a reduction in DNA damage and lipid oxidation.

In addition, greater maternal DASH score was related to decreased levels of maternal urinary 15-F2t-isoprostane measured at mid-pregnancy. The DASH diet is a diet high in fruits, vegetables, total grains, nuts, seeds, legumes, and non-full-fat dairy products and low in animal protein, sugar, and sodium. Our results are consistent with previous findings reported in adults suggesting that a long-term adherence to a diet rich in fruits and vegetables and low in red meat may decrease lipid peroxidation measured as the plasma levels of F2-isoprostanes (44). These findings support the protective effects of a diet high in fruits, vegetables, nuts, seeds, and whole grains, and low in red and processed meats and refined grains lipid peroxidation in pregnant women. The DASH diet has been shown to reduce blood pressure, a lower risk of cardiovascular disease, improve insulin sensitivity, and aid in weight loss in non-pregnant populations. Although some studies have failed to find an association between DASH diet and hypertensive disorder and GDM (45, 46), others have reported positive associations. Asemi et al. showed that adherence to the DASH diet beginning at 24–28 weeks gestation reduced systolic blood pressure and plasma glucose, and improved lipid profile during the 4-week study period (47, 48). Similarly, analysis of NHS II data revealed that adherence to several healthful dietary patterns, such as the DASH diet, prior to pregnancy corresponded with a lower risk of GDM (49). Some of the pathophysiologic changes occurring in these conditions developed during pregnancy are related to oxidative stress, and the components of the DASH diet may reduce maternal oxidative stress and enhance antioxidant capacity, which could result in reduced related pregnancy complications, such as hypertension disorders, insulin resistance, metabolic syndrome, and GDM (4,5).

Regarding the AHEI scores, we found greater maternal AHEI score to be related with the reduced offspring urinary 15-F2t-isoprostane levels. Although not statistically significant, a similar trend was observed for maternal urinary 15-F2t-isoprostane levels measured at mid-pregnancy. Our results are consistent with previous studies in adolescents and adults showing that a long-term adherence to a diet rich in fruits and vegetables and low in red meat may decrease lipid peroxidation (44, 50).

Our study had several strengths, such as its population-based, prospective design, the use of validated FFQ to evaluate diet and strict protocols, and the availability of extensive information on potential confounders. Some study weaknesses also deserve mention. In some instances, we might have had limited statistical power to detect associations within our cohort. Maternal dietary habits were evaluated using a FFQ, subjected to measurement errors that may led to an attenuation of effect estimates. Nevertheless, the FFQ employed in this work was previously validated in pregnant women of the same area (19). We applied dietary indices which were not specifically created for the Spanish population, except the rMED index that was implemented for the Spanish cohort of European Prospective Investigation into Cancer (EPIC). Our analysis was limited to a single measure of biomarkers of oxidative stress.

In conclusion, our findings provide the first evidence that maternal adherence to healthy dietary patterns during pregnancy (i.e., rMED, DASH, and AHEI) may reduce DNA damage and lipid oxidation in mothers and offspring. Changes in the biomarkers of oxidative stress in relation to maternal adherence to dietary patterns in pregnancy could mediate the reported effects of maternal dietary patterns on pregnancy outcomes and offspring fetal development.

## NELA Study Group

Biomedical Research Institute of Murcia, IMIB-Arrixaca, Murcia, Spain: ME Candel-Torralba, L Garcia-Marcos (PI), MJ Gimenez-Banon, A Martinez-Torres, E Morales (PI), V Perez-Fernandez, M Sanchez-Solis, A Nieto, MT Prieto-Sanchez, M Sanchez-Ferrer, L Fernanez-Palacios, VP Gomez-Gomez, C Martinez-Gracia, P Peso-Echarri, G Ros-Berruezo, M Santaella-Pacual, A Gazquez, E Larque, MT Pastor-Fajardo, M Sanchez-Campillo, A Serrano-Munuera, M Zornoza-Moreno, P Jimenez-Guerrero, E Adomnei, JJ Arense-Gonzalo, J Mendiola, F Navarro-Lafuente, AM Torres-Cantero, M Segovia-Hernández, G Yagüe-Guirao, PL Valero-Guillén, FV Aviles-Plaza, J Cabezas-Herrera, A Martinez-Lopez, M Martinez-Villanueva, JA Noguera-Velasco, A Franco-Garcia, AM Garcia-Serna, T Hernandez-Caselles, E Martin-Orozco, M Norte-Muñoz, M Canovas, E Cantero-Cano, T de Diego, JM Pastor, RA Sola-Martínez, A Esteban-Gil, JT Fernández-Breis. Paediatric Respiratory Unit, “Virgen de la Arrixaca” Children's University Clinical Hospital, University of Murcia, Spain: L Garcia-Marcos (PI), A Martinez-Torres, M Sanchez-Solis. Department of Public Health Sciences, University of Murcia, Murcia, Spain: E Morales (PI). Department of Pediatrics, University of Murcia, Murcia, Spain: L Garcia-Marcos (PI), V Perez-Fernandez, M Sanchez-Solis. Obstetrics & Gynecology Service, “Virgen de la Arrixaca” University Clinical Hospital, University of Murcia, Murcia, Spain: A Nieto, MT Prieto-Sanchez, M Sanchez-Ferrer. Food Science and Technology Department, Veterinary Faculty of Veterinary, University of Murcia, Spain: L Fernanez-Palacios, VP Gomez-Gomez, C Martinez-Gracia, P Peso-Echarri, G Ros-Berruezo, M Santaella-Pacual. Department of Physiology, Faculty of Biology, Campus Mare Nostrum. University of Murcia, Spain: A Gazquez, E Larque, MT Pastor-Fajardo, M Sanchez-Campillo, A Serrano-Munuera, M Zornoza-Moreno. Regional Atmospheric Modelling Group, Department of Physics, University of Murcia, Murcia, Spain: P Jimenez-Guerrero. Department of Public Health Sciences, University of Murcia, Murcia, Spain: E Adomnei, JJ Arense-Gonzalo, J Mendiola, F Navarro-Lafuente, AM Torres-Cantero. Microbiology Service, General University Hospital Consortium, University of Valencia, Valencia, Spain: C Salvador-Garcia. Microbiology Service, University Clinical Hospital “Virgen de la Arrixaca”, University of Murcia, Murcia, Spain: M Segovia-Hernández, G Yagüe-Guirao. Microbiology and Genetics Department, University of Murcia, Murcia, Spain: PL Valero-Guillén. Molecular Therapy and Biomarkers Research Group, Clinical Analysis Service, University Clinical Hospital “Virgen de la Arrixaca”, University of Murcia, Murcia, Spain: FV Aviles-Plaza, J Cabezas-Herrera, A Martinez-Lopez, M Martinez-Villanueva, JA Noguera-Velasco. Department of Biochemistry and Molecular Biology B and Immunology, University of Murcia, Murcia, Spain: A Franco-Garcia, AM Garcia-Serna, T Hernandez-Caselles, E Martin-Orozco, M Norte-Muñoz, M Canovas, T de Diego, JM Pastor, RA Sola-Martínez. Department of Informatics and Systems, University of Murcia, Murcia, Spain: JT Fernández-Breis. Paediatric and Adolescent Clinical Psychology University Research Group (GUIIA-PC), University of Murcia, Murcia, Spain: MV Alcántara, S Hernández, C López-Soler. Foundation for Healthcare Training & Research of the Region of Murcia (FFIS), Murcia, Spain: A Esteban-Gil. Network of Asthma and Adverse and Allergic Reactions (ARADyAL), Madrid, Spain: L Garcia-Marcos (PI), A Martinez-Torres, V Perez-Fernandez, M Sanchez-Solis, T Hernandez-Caselles, E Martin-Orozco

## Data Availability Statement

Data described in the article, code book, and analytic code will be made available upon request pending application and approval of the NELA Steering Committee. Requests to access the datasets should be directed to evamorales@um.es.

## Ethics Statement

The studies involving human participants were reviewed and approved by Ethics Committee of the Virgen de la Arrixaca University Clinical Hospital (report 9/14; 29/09/2014). Written informed consent to participate in this study was provided by the participants' legal guardian/next of kin.

## Author Contributions

EM and LG-M conceived and supervised the project and recruited the participants. CM-G, MS-P, CS-M, and JV collected and provided dietary data. CB-M and LG-C collected the biological samples. AG-S, EL, MS-C, AS-M, JN-V, FA-P, and MM-V designed and performed the experiments. EM conceived the study, conducted the statistical analyses, and wrote the article. All authors have reviewed the results and have critically revised the final version of the manuscript.

## Funding

This study was supported by grants from the Instituto de Salud Carlos III, Spanish Ministry of Science, Innovation and Universities, and co-funded by the European Union (Grant Numbers: CP14/00046, PIE15/00051, PI16/00422, PI19/00863, and ARADyAL network RD160006). AG-S was funded by a predoctoral Fellowship (FI17/00086) and EM was funded by Miguel Servet Fellowships (MS14/00046 and CPII19/00019) awarded by the Instituto de Salud Carlos III (ISCIII), Spanish Ministry of Science, Innovation and Universities, and Fondos FEDER. The funders had no role in study design, data collection and analysis, decision to publish, or preparation of the manuscript.

## Conflict of Interest

The authors declare that the research was conducted in the absence of any commercial or financial relationships that could be construed as a potential conflict of interest.

## Publisher's Note

All claims expressed in this article are solely those of the authors and do not necessarily represent those of their affiliated organizations, or those of the publisher, the editors and the reviewers. Any product that may be evaluated in this article, or claim that may be made by its manufacturer, is not guaranteed or endorsed by the publisher.
