# Supplementary material for: Dietary Patterns in Pregnancy and Biomarkers of Oxidative Stress in Mothers and Offspring: The NELA Birth Cohort
Source: Front Nutr. 2022 Apr 12;9:869357. doi: 10.3389/fnut.2022.869357 (PMC9039535; doi:10.3389/fnut.2022.869357)
Supplement: Supplementary file 1 [file Data_Sheet_1.docx]

Supplementary Material

**Dietary patterns in pregnancy and biomarkers of oxidative stress in mothers and offspring: the NELA birth cohort**

**Supplementary Figure 1.** Flow-chart of the study population.


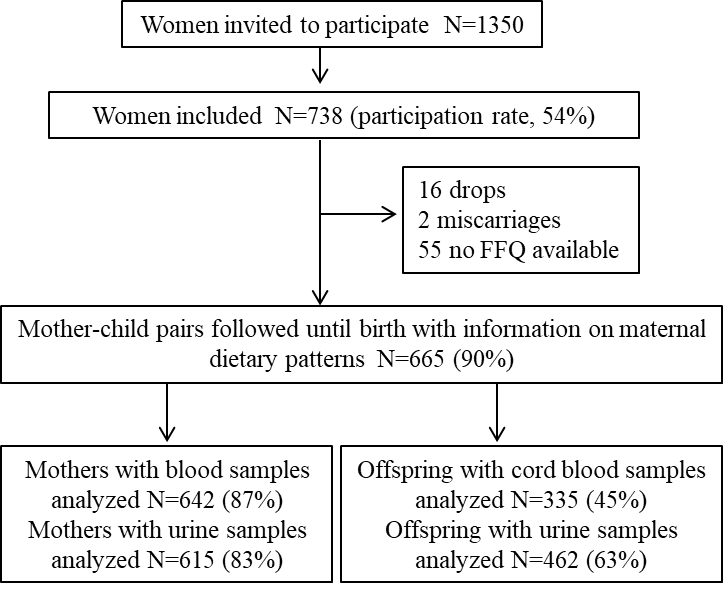


**Supplementary Table 1.** Description of scoring system and components of maternal dietary indices during pregnancy. The NELA study.

|  | **rMED** | **aMED** | **DASH** | **AHEI** | **AHEI-2010** |
| --- | --- | --- | --- | --- | --- |
| **Scoring system** | Tertiles (T) of grams per 1000 kcal/day:  0-2 points | Median intake: 0-1 points | Quintiles (Q): 1-5 points | Pre-defined cut-offs: 0-10 points | Pre-defined cut-offs: 0-10 points |
| **Component** |  |  |  |  |  |
| Vegetables | T3: 2 points | > Median intake: 1 point | Q5: 5 points | 5 servings/d: 10 points; 1 point less for each 10% less than intake required for full score | 5 servings/d: 10 points; 1 point less for each 10% less than intake required for full score |
| Fruit | T3 (including nuts): 2 points | > Median intake: 1 point | Q5: 5 points | 4 servings/d: 10 points; 1 point less for each 10% less than intake required for full score | 4 servings/d: 10 points; 1 point less for each 10% less than intake required for full score |
| Nuts | - | > Median intake: 1 point | - | 1 servings/d: 10 points; 1 point less for each 10% less than intake required for full score | - |
| Nuts and legumes |  | - | Q5: 5 points | - | 1 servings/d: 10 points; 1 point less for each 10% less than intake required for full score |
| Legumes | T3: 2 points | > Median intake: 1 point |  | - | - |
| Cereals | - | - | - | - | - |
| Whole grains | T3 (including refined grains): 2 points | > Median intake: 1 point | Q5: 5 points | 10 points; 1 point less for each 10% less than intake required for full score | 75g/d: 10 points |
| Cereal fiber | - | - | - | 15 g/day; 1 point less for each 10% less than intake required for full score | - |
| Meat | - | - | - | - |  |
| Red and processed meat | T1 (including white meat): 2 points | < Median intake: 1 point | Q1: 5 points | - | ≥1.5 servings/d: 0 points |
| Ratio of white to red meat |  | - | - | 4: 10 points; 1 point less for each 10% less than intake required for full score |  |
| Fish | T3: 2 points | > Median intake: 1 point | - | - |  |
| Dairy products | T1: 2 points | - | Q5 (only low-fat): 5 points | - |  |
| Olive oil | T3: 2 points | - | - | - |  |
| Sugar-sweetened beverages and fruit juices | - | - | Q1: 5 points | - | ≥1 serving/d: 0 points |
| Alcohol |  |  | - | 0.5-1.5 servings/day: 10 points  Intake<”ideal”: 1 point less for each 10% than ideal intake  Intake> “ideal”: 1 point less for each 10% above than ideal intake  0 or >2.5 servings/d: 0 points | 0.5-1.5 drinks/d: 10 points  ≥2.5 drinks/d: 0 points |
| Sodium | - | - | Q1: 5 points | - | Lowest decile in mg/d: 10 points |
| Ratio of monosaturated to saturated fats | - | > Median intake: 1 point | - | - |  |
| Ratio of polyunsaturated to saturated | - |  | - | ≥1: 10 points; 1 point less for each 10% less than intake required for full score | - |
| Trans fat | - | - | - | ≤0.5% of energy: 10 points  >0.5 but 4.0% of energy: 1 point less for each 10% increment in this range  ≥4: 0 points | ≤0.5% of energy: 10 points  >0.5 but 4.0% of energy: 1 point less for each 10% increment in this range  ≥4: 0 points |
| Polyunsaturated | - | - | - | - | ≥10% of energy: 10 points  ≤2% of energy: 0 points |
| w3 fats (EPA + DHA) | - | - | - | - | ≥250 mg: 10 points |
| **Total maximum score** | 16 | 8 | 40 | 80 | 110 |

**Supplementary Table 2**. Spearman’s correlations coefficients of maternal dietary scores during pregnancy. The NELA study.

|  | **rMED** | **aMED** | **DASH** | **AHEI** | **AHEI-2010** |
| --- | --- | --- | --- | --- | --- |
| **rMED** | 1.00 |  |  |  |  |
| **aMED** | 0.61 | 1.00 |  |  |  |
| **DASH** | 0.53 | 0.71 | 1.00 |  |  |
| **AHEI** | 0.52 | 0.76 | 0.64 | 1.00 |  |
| **AHEI-2010** | 0.38 | 0.30 | 0.55 | 0.23 | 1.00 |

rMED: Relative Mediterranean Diet Score; aMED: Alternate Mediterranean Diet; DASH: Dietary Approach to Stop Hypertension; AHEI: Alternate Healthy Eating Index; AHEI-2010: Alternate Healthy Eating Index 2010.

**Supplementary Table 3**. Relationship between baseline characteristics and maternal Relative Mediterranean Diet (rMED) score in mid-pregnancy. The NELA study.

|  |  | **Tertiles of maternal rMED score** | | |  |
| --- | --- | --- | --- | --- | --- |
|  | **n** | **T1 (n=288)** | **T2 (n=185)** | **T3 (n=192)** | **P value** |
| Maternal age, mean (sd) | 665 | 31.6 (4.6) | 33.1 (4.2) | 34.3 (4.2) | <0.001 |
| Maternal education level, % | 665 |  |  |  |  |
| Incomplete secondary or less |  | 24.0 | 17.8 | 12.0 | <0.001 |
| Complete secondary |  | 29.9 | 24.9 | 19.8 |  |
| University |  | 46.2 | 57.3 | 68.2 |  |
| Maternal social class, % | 665 |  |  |  |  |
| I-II |  | 31.3 | 40.5 | 43.8 | 0.007 |
| III |  | 21.9 | 18.4 | 25.5 |  |
| IV-V |  | 21.2 | 23.2 | 14.1 |  |
| Unemployed |  | 25.7 | 17.8 | 16.7 |  |
| BMI before pregnancy (kg/m^2^), mean (sd) | 661 | 24.1 (4.7) | 23.7 (4.1) | 24.0 (4.8) | 0.551 |
| 18-24.99 |  | 68.3 | 70.1 | 68.9 | 0.939 |
| 25-29.99 |  | 22.3 | 22.3 | 21.1 |  |
| 30+ |  | 9.4 | 7.6 | 10.0 |  |
| Parity, nulliparous, % | 665 | 48.6 | 53.5 | 47.4 | 0.445 |
| Maternal smoking in pregnancy, yes, % | 665 | 20.1 | 15.1 | 10.9 | 0.025 |
| Physical activity in pregnancy | 665 |  |  |  |  |
| Sedentary |  | 17.0 | 16.2 | 14.6 | 0.442 |
| Poorly active |  | 47.6 | 39.5 | 43.2 |  |
| Moderately active |  | 32.3 | 38.9 | 36.5 |  |
| Strongly active |  | 3.1 | 5.4 | 5.7 |  |
| Paracetamol use in pregnancy, ever, % | 622 | 69.5 | 67.6 | 52.0 | <0.001 |
| Gestational DM, yes, % | 649 | 6.5 | 5.5 | 13.2 | 0.010 |
| Hypertension in pregnancy, yes, % | 644 | 2.9 | 2.2 | 2.1 | 0.860 |
| Gestational weight gain, mean (sd) | 619 | 12.5 (4.7) | 12.0 (5.0) | 11.3 (5.0) | 0.044 |
| Type of delivery, (%) |  |  |  |  |  |
| Vaginal non-instrumental | 649 | 59.3 | 55.0 | 59.3 | 0.444 |
| Vaginal Instrumental |  | 22.1 | 19.4 | 21.7 |  |
| Caesarean section |  | 18.6 | 25.6 | 19.0 |  |
| Oxytocin use, yes, (%) | 565 | 80.1 | 76.1 | 73.8 | 0.309 |
| Newborn sex, male, (%) | 654 | 45.8 | 52.8 | 52.1 | 0.239 |
| Apgar score (5th min), mean (sd) | 647 | 9.92 (0.42) | 9.91 (0.43) | 9.99 (0.10) | 0.060 |
| Gestational age, mean (sd) | 654 | 39.6 (1.5) | 39.6 (1.5) | 39.6 (1.4) | 0.867 |
| Birthweight, mean (sd) | 649 | 3242 (477) | 3238 (443) | 3261 (457) | 0.878 |
| Season of birth, % | 654 |  |  |  |  |
| Autumn |  | 29.6 | 26.1 | 28.9 | 0.469 |
| Spring |  | 27.1 | 24.4 | 22.1 |  |
| Summer |  | 29.2 | 28.3 | 32.6 |  |
| Winter |  | 14.1 | 21.1 | 16.3 |  |
| Calories intake (kcal/day) | 665 | 2266.6 (770.0) | 2070.4 (485.8) | 2014.4 (522.6) | <0.001 |
| Alcohol intake (g/day) | 665 | 0.16 (0.39) | 0.20 (0.77) | 0.14 (0.31) | 0.430 |

**Supplementary Table 4**. Relationship between baseline characteristics and maternal Alternate Mediterranean Diet (aMED) score in mid-pregnancy. The NELA study.

|  |  | **Tertiles of maternal aMED score** | | |  |
| --- | --- | --- | --- | --- | --- |
|  | **n** | **T1 (n=290)** | **T2 (n=245)** | **T3 (n=130)** | **P value** |
| Maternal age, mean (sd) | 665 | 31.8 (4.8) | 33.2 (4.1) | 34.2 (4.1) | <0.001 |
| Maternal education level, % | 665 |  |  |  |  |
| Incomplete secondary or less |  | 26.9 | 14.7 | 8.5 | <0.001 |
| Complete secondary |  | 28.6 | 24.5 | 20.8 |  |
| University |  | 44.5 | 60.8 | 70.8 |  |
| Maternal social class, % | 665 |  |  |  |  |
| I-II |  | 29.7 | 39.6 | 50.8 | <0.001 |
| III |  | 20.3 | 23.7 | 22.3 |  |
| IV-V |  | 24.1 | 18.8 | 11.5 |  |
| Unemployed |  | 25.9 | 17.9 | 15.4 |  |
| BMI before pregnancy (kg/m^2^), mean (sd) | 661 | 24.3 (4.7) | 23.7 (4.4) | 23.7 (4.5) | 0.227 |
| Parity, nulliparous, % | 665 | 44.1 | 52.7 | 56.2 | 0.037 |
| Maternal smoking in pregnancy, yes, % | 665 | 19.3 | 17.1 | 6.9 | 0.005 |
| Physical activity in pregnancy | 665 |  |  |  |  |
| Sedentary |  | 18.3 | 15.9 | 11.5 | 0.014 |
| Poorly active |  | 49.0 | 40.4 | 40.0 |  |
| Moderately active |  | 30.0 | 39.2 | 40.0 |  |
| Strongly active |  | 2.7 | 4.5 | 8.5 |  |
| Paracetamol use in pregnancy, ever, % | 622 | 70.9 | 61.8 | 52.9 | 0.002 |
| Gestational DM, yes, % | 649 | 7.9 | 5.8 | 13.2 | 0.047 |
| Hypertension in pregnancy, yes, % | 644 | 2.5 | 2.9 | 1.6 | 0.717 |
| Gestational weight gain, mean (sd) | 619 | 12.1 (4.8) | 12.3 (4.7) | 11.2 (5.4) | 0.121 |
| Type of delivery, (%) | 649 |  |  |  |  |
| Vaginal non-instrumental |  | 62.3 | 56.9 | 51.2 | 0.230 |
| Vaginal Instrumental |  | 18.9 | 20.9 | 27.1 |  |
| Caesarean section |  | 18.9 | 22.2 | 21.7 |  |
| Oxytocin use, yes, (%) | 565 | 77.1 | 78.5 | 74.8 | 0.755 |
| Newborn sex, male, (%) | 654 | 46.3 | 49.8 | 56.2 | 0.176 |
| Apgar score at 5 min, mean (sd) | 647 | 9.93(0.34) | 9.93 (0.43) | 9.96 (0.23) | 0.700 |
| Gestational age, mean (sd) | 654 | 39.7 (1.4) | 39.5 (1.5) | 39.5 (1.6) | 0.179 |
| Birthweight, mean (sd) | 649 | 3251.5 (453.8) | 3217.9 (453.5) | 3290.9 (493.2) | 0.343 |
| Season of birth, (%) | 654 |  |  |  |  |
| Autumn |  | 30.7 | 27.0 | 26.2 | 0.657 |
| Spring |  | 24.0 | 23.7 | 29.2 |  |
| Summer |  | 27.9 | 31.5 | 31.5 |  |
| Winter |  | 17.3 | 17.8 | 13.1 |  |
| Calories intake (kcal/day) | 665 | 2062 (674.5) | 2166.6 (629.7) | 2257 (573.1) | 0.011 |
| Alcohol intake (g/day) | 665 | 0.15 (0.55) | 0.18 (0.50) | 0.17 (0.42) | 0.699 |

**Supplementary Table S5**. Relationship between baseline characteristics and maternal Dietary Approach to Stop Hypertension (DASH) score in mid-pregnancy. The NELA study.

|  |  | **Tertiles of maternal DASH score** | | |  |
| --- | --- | --- | --- | --- | --- |
|  | **n** | **T1 (n=233)** | **T2 (n=217)** | **T3 (n=215)** | **P value** |
| Maternal age, mean (sd) | 665 | 31.3 (5.1) | 33.1 (4.0) | 34.1 (3.8) | <0.001 |
| Maternal education level, % | 665 |  |  |  |  |
| Incomplete secondary or less |  | 31.8 | 14.7 | 8.8 | <0.001 |
| Complete secondary |  | 30.9 | 24.9 | 20.5 |  |
| University |  | 37.3 | 60.4 | 70.7 |  |
| Maternal social class, % | 665 |  |  |  |  |
| I-II |  | 21.0 | 41.5 | 51.2 | <0.001 |
| III |  | 23.6 | 18.0 | 24.2 |  |
| IV-V |  | 27.0 | 21.7 | 9.8 |  |
| Unemployed |  | 28.3 | 18.9 | 14.9 |  |
| BMI before pregnancy (kg/m^2^), mean (sd) | 661 | 24.5 (5.1) | 23.6 (4.1) | 23.8 (4.4) | 0.116 |
| Parity, nulliparous, % | 665 | 42.9 | 50.2 | 56.3 | 0.018 |
| Maternal smoking in pregnancy, yes, % | 665 | 24.0 | 13.8 | 9.8 | <0.001 |
| Physical activity in pregnancy | 665 |  |  |  |  |
| Sedentary |  | 18.9 | 14.8 | 14.4 | 0.002 |
| Poorly active |  | 48.9 | 44.2 | 38.6 |  |
| Moderately active |  | 30.5 | 37.8 | 38.1 |  |
| Strongly active |  | 1.7 | 3.2 | 8.8 |  |
| Paracetamol use in pregnancy, ever, % | 622 | 73.6 | 62.9 | 55.1 | <0.001 |
| Gestational DM, yes, % | 649 | 9.4 | 4.7 | 10.4 | 0.069 |
| Hypertension in pregnancy, yes, % | 644 | 2.2 | 3.8 | 1.4 | 0.293 |
| Gestational weight gain, mean (sd) | 619 | 12.2 (5.1) | 12.2 (4.9) | 11.6 (4.7) | 0.376 |
| Type of delivery, (%) | 649 |  |  |  |  |
| Vaginal non-instrumental |  | 59.6 | 57.8 | 56.9 | 0.713 |
| Vaginal Instrumental |  | 18.2 | 23.0 | 22.7 |  |
| Caesarean section |  | 22.2 | 19.2 | 20.4 |  |
| Oxytocin use, yes, (%) | 565 | 74.9 | 79.2 | 77.7 | 0.594 |
| Newborn sex, male, (%) | 654 | 47.6 | 45.3 | 55.9 | 0.071 |
| Apgar score at 5 min, mean (sd) | 647 | 9.93 (0.30) | 9.91 (0.52) | 0.97 (0.19) | 0.172 |
| Gestational age, mean (sd) | 654 | 39.7 (1.5) | 39.5 (1.5) | 39.6 (1.5) | 0.665 |
| Birthweight, mean (sd) | 649 | 3275 (475) | 3187 (452) | 3276 (453) | 0.073 |
| Season of birth, (%) | 654 |  |  |  |  |
| Autumn |  | 30.0 | 27.6 | 27.7 | 0.801 |
| Spring |  | 22.9 | 23.8 | 28.2 |  |
| Summer |  | 28.6 | 31.8 | 29.6 |  |
| Winter |  | 15.8 | 16.8 | 14.5 |  |
| Calories intake (kcal/day), mean (sd) | 665 | 2289.7 (763.2) | 2099.7 (618.6) | 2016.0 (471.7) | <0.001 |
| Alcohol intake (g/day), mean (sd) | 665 | 0.17 (0.62) | 0.16 (0.34) | 0.17 (0.52) | 0.966 |

**Supplementary Table 6**. Relationship between baseline characteristics and maternal Alternate Healthy Eating Index (AHEI) score in mid-pregnancy. The NELA study.

|  |  | **Tertiles of maternal AHEI score** | | |  |
| --- | --- | --- | --- | --- | --- |
|  | **n** | **T1 (n=235)** | **T2 (n=211)** | **T3 (n=219)** | **P value** |
| Maternal age, mean (± sd) | 665 | 31.8 (4.9) | 32.9 (4.3) | 33.8 (4.1) | <0.001 |
| Maternal education level, % | 665 |  |  |  |  |
| Incomplete secondary or less |  | 24.2 | 19.4 | 12.4 | 0.005 |
| Complete secondary |  | 26.4 | 27.5 | 22.8 |  |
| University |  | 49.4 | 53.1 | 64.8 |  |
| Maternal social class, % | 665 |  |  |  |  |
| I-II |  | 32.3 | 34.1 | 46.1 | 0.013 |
| III |  | 20.9 | 21.3 | 23.7 |  |
| IV-V |  | 22.1 | 21.8 | 15.1 |  |
| Unemployed |  | 24.7 | 22.8 | 15.1 |  |
| BMI before pregnancy (kg/m^2^), mean (± sd) | 661 | 24.0 (4.3) | 24.1 (1.9) | 23.9 (4.5) | 0.875 |
| Parity, nulliparous, % | 665 | 45.9 | 49.3 | 53.9 | 0.239 |
| Maternal smoking in pregnancy, yes, % | 665 | 17.5 | 16.6 | 14.2 | 0.617 |
| Physical activity in pregnancy | 665 |  |  |  |  |
| Sedentary |  | 17.0 | 16.1 | 15.1 | 0.002 |
| Poorly active |  | 51.9 | 42.2 | 37.4 |  |
| Moderately active |  | 28.9 | 38.4 | 39.3 |  |
| Strongly active |  | 2.1 | 3.3 | 8.2 |  |
| Paracetamol use in pregnancy, ever, % | 622 | 68.9 | 62.7 | 60.2 | 0.163 |
| Gestational DM, yes, % | 649 | 7.9 | 6.7 | 9.8 | 0.506 |
| Hypertension in pregnancy, yes, % | 644 | 3.1 | 1.9 | 2.3 | 0.723 |
| Gestational weight gain, mean (sd) | 619 | 12.3 (4.7) | 12.1 (5.0) | 11.6 (5.0) | 0.287 |
| Type of delivery, (%) | 649 |  |  |  |  |
| Vaginal non-instrumental |  | 59.5 | 59.2 | 55.6 | 0.689 |
| Vaginal Instrumental |  | 18.9 | 20.4 | 24.5 |  |
| Caesarean section |  | 21.6 | 20.4 | 19.9 |  |
| Oxytocin use, yes, (%) | 565 | 79.2 | 75.0 | 77.1 | 0.613 |
| Newborn sex, male, (%) | 654 | 45.2 | 49.8 | 53.9 | 0.182 |
| Apgar score at 5 min, mean (± sd) | 647 | 9.9 (0.4) | 9.9 (0.4) | 9.9 (0.3) | 0.916 |
| Gestational age, mean (± sd) | 654 | 39.7 (1.4) | 39.6 (1.5) | 39.5 (1.6) | 0.399 |
| Birthweight (g), mean (± sd) | 649 | 3270.5 (486.6) | 3238.7 (409.2) | 3229.7 (483.1) | 0.621 |
| Season of birth, (%) | 654 |  |  |  |  |
| Autumn |  | 32.5 | 25.8 | 26.7 | 0.190 |
| Spring |  | 21.9 | 25.8 | 27.2 |  |
| Summer |  | 28.1 | 28.2 | 33.6 |  |
| Winter |  | 17.5 | 20.1 | 12.4 |  |
| Calories intake (kcal/day), mean (sd) | 665 | 2016.8 (681.7) | 2114.8 (563.9) | 2294.1 (642.1) | <0.001 |
| Alcohol intake (g/day), mean (sd) | 665 | 0.14 (0.33) | 0.16 (0.60) | 0.20 (0.57) | 0.460 |

**Supplementary Table 7**. Relationship between baseline characteristics and maternal Alternate Healthy Eating Index 2010 (AHEI-2010) score in mid-pregnancy. The NELA study.

|  |  | **Tertiles of maternal AHEI-2010 score** | | |  |
| --- | --- | --- | --- | --- | --- |
|  | **n** | **T1 (n=259)** | **T2 (n=186)** | **T3 (n=220)** | **P value** |
| Maternal age, mean (± sd) | 665 | 31.4 (4.9) | 33.6 (4.0) | 33.7 (4.0) | <0.001 |
| Maternal education level, % | 665 |  |  |  |  |
| Incomplete secondary or less |  | 24.7 | 12.9 | 16.8 | 0.001 |
| Complete secondary |  | 28.2 | 29.0 | 19.6 |  |
| University |  | 47.1 | 58.1 | 63.6 |  |
| Maternal social class, % | 665 |  |  |  |  |
| I-II |  | 29.7 | 41.9 | 42.7 | 0.007 |
| III |  | 22.8 | 21.0 | 21.8 |  |
| IV-V |  | 25.9 | 13.4 | 17.7 |  |
| Unemployed |  | 21.6 | 23.7 | 17.7 |  |
| BMI before pregnancy (kg/m^2^), mean (± sd) | 661 | 23.8 (4.4) | 24.1 (4.3) | 24.1 (4.9) | 0.715 |
| Parity, nulliparous, % | 665 | 55.6 | 48.4 | 43.6 | 0.031 |
| Maternal smoking in pregnancy, yes, % | 665 | 22.4 | 12.9 | 11.4 | 0.002 |
| Physical activity in pregnancy | 665 |  |  |  |  |
| Sedentary |  | 17.4 | 14.5 | 15.9 | 0.117 |
| Poorly active |  | 44.8 | 48.4 | 39.6 |  |
| Moderately active |  | 35.9 | 31.7 | 37.7 |  |
| Strongly active |  | 1.9 | 5.4 | 6.8 |  |
| Paracetamol use in pregnancy, ever, % | 622 | 66.1 | 67.1 | 58.9 | 0.176 |
| Gestational DM, yes, % | 649 | 8.0 | 10.4 | 6.5 | 0.353 |
| Hypertension in pregnancy, yes, % | 644 | 2.4 | 3.9 | 1.4 | 0.301 |
| Gestational weight gain, mean (sd) | 619 | 12.9 (5.0) | 11.5 (4.8) | 11.4 (4.8) | 0.002 |
| Type of delivery, (%) | 649 |  |  |  |  |
| Vaginal non-instrumental |  | 54.0 | 55.7 | 64.9 | 0.160 |
| Vaginal Instrumental |  | 23.0 | 23.5 | 17.3 |  |
| Caesarean section |  | 23.0 | 20.8 | 17.8 |  |
| Oxytocin use, yes, (%) | 565 | 76.6 | 83.7 | 72.6 | 0.052 |
| Newborn sex, male, (%) | 654 | 51.0 | 47.3 | 49.8 | 0.744 |
| Apgar score at 5 min, mean (± sd) | 647 | 9.9 (0.4) | 9.9 (0.3) | 9.9 (0.4) | 0.915 |
| Gestational age, mean (± sd) | 654 | 39.6 (1.5) | 39.5 (1.6) | 39.7 (1.4) | 0.500 |
| Birthweight (g), mean (± sd) | 649 | 3228.6 (446.9) | 3205.7 (511.6) | 3303.4 (429.5) | 0.080 |
| Season of birth, (%) | 654 |  |  |  |  |
| Autumn |  | 29.0 | 30.4 | 26.1 | 0.597 |
| Spring |  | 23.1 | 22.3 | 29.3 |  |
| Summer |  | 31.8 | 28.3 | 29.3 |  |
| Winter |  | 16.1 | 19.0 | 15.3 |  |
| Calories intake (kcal/day), mean (sd) | 665 | 2360.8 (693.8) | 2053.3 (670.1) | 1950.9 (454.2) | <0.001 |
| Alcohol intake (g/day), mean (sd) | 665 | 0.18 (0.42) | 0.16 (0.46) | 0.15 (0.63) | 0.801 |
